# MicroRNA-301a Mediated Regulation of Kv4.2 in Diabetes: Identification of Key Modulators

**DOI:** 10.1371/journal.pone.0060545

**Published:** 2013-04-03

**Authors:** Siva K. Panguluri, Jared Tur, Kalyan C. Chapalamadugu, Chris Katnik, Javier Cuevas, Srinivas M. Tipparaju

**Affiliations:** 1 Department of Pharmaceutical Sciences, University of South Florida - College of Pharmacy, Tampa, Florida, United States of America; 2 Department of Molecular Pharmacology and Physiology, University of South Florida – Morsani College of Medicine, Tampa, Florida, United States of America; 3 Department of Molecular Medicine, University of South Florida – Morsani College of Medicine, Tampa, Florida, United States of America; Boston University School of Medicine, United States of America

## Abstract

Diabetes is a metabolic disorder that ultimately results in major pathophysiological complications in the cardiovascular system. Diabetics are predisposed to higher incidences of sudden cardiac deaths (SCD). Several studies have associated diabetes as a major underlying risk for heart diseases and its complications. The diabetic heart undergoes remodeling to cope up with the underlying changes, however ultimately fails. In the present study we investigated the changes associated with a key ion channel and transcriptional factors in a diabetic heart model. In the mouse db/db model, we identified key transcriptional regulators and mediators that play important roles in the regulation of ion channel expression. Voltage-gated potassium channel (Kv4.2) is modulated in diabetes and is down regulated. We hypothesized that Kv4.2 expression is altered by potassium channel interacting protein-2 (KChIP2) which is regulated upstream by NFkB and miR-301a. We utilized qRT-PCR analysis and identified the genes that are affected in diabetes in a regional specific manner in the heart. At protein level we identified and validated differential expression of Kv4.2 and KChIP2 along with NFkB in both ventricles of diabetic hearts. In addition, we identified up-regulation of miR-301a in diabetic ventricles. We utilized loss and gain of function approaches to identify and validate the role of miR-301a in regulating Kv4.2. Based on *in vivo* and *in vitro* studies we conclude that miR-301a may be a central regulator for the expression of Kv4.2 in diabetes. This miR-301 mediated regulation of Kv4.2 is independent of NFkB and Irx5 and modulates Kv4.2 by direct binding on Kv4.2 3′untranslated region (3′-UTR). Therefore targeting miR-301a may offer new potential for developing therapeutic approaches.

## Introduction

Diabetes mellitus (DM) is a chronic and major disease affecting a large population in US and across the world. Diabetic cardiomyopathy (DCM) accounts for 70% deaths among diabetic patients. DCM leads to the inability of the heart to circulate blood efficiently and the disease progression is mostly asymptomatic until late stages. Current understanding in this area indicates that diabetics have increased incidences of sudden cardiac death due to myocardial ischemia (MI), when compared to non-diabetics [1], [2]. The heart is a highly metabolic organ which functions as a synchronized electrical unit for pumping of oxygenated blood to the body. However in diabetes, the electrical activity is compromised and predisposes the heart to maladaptation and metabolic disturbances.

Based on the existing literature, it is evident that electrical remodeling occurs as a consequence of diabetes and potassium channels (Kv) play a major role in the consequential DCM [3], [4]. Potassium channels are known to regulate the shape and duration of the action potential, which in turn governs the function of the heart. The outward potassium currents regulate the membrane potential and the action potential duration. Among the various members of potassium channel family (Kv1-12), the Kv4.2 (Voltage gate potassium channel: Kv4.2) is the underlying ion channel that helps the heart maintain repolarization reserve.

Many laboratories have identified potential mechanism(s) responsible for electrical remodeling in diabetic hearts [4], [5]. Nonetheless, the underlying molecular mechanisms remain unclear. Therefore, in the present study we sought to elucidate the molecular basis of diabetic arrhythmogenesis from the perspective of repolarization reserve. The diabetic heart undergoes remodeling in a manner that disrupts electrical synchrony, predisposing the heart to electrical abnormalities and arrhythmias. Normal cardiac function requires large amounts of ATP, and generation of adequate amounts of this high energy phosphate molecule is dependent on optimal performance of various metabolic signaling pathways that process energy substrates. Systemic changes in these metabolic pathways, which commonly occur in diabetes, can significantly impair cardiac efficiency and decrease energy generation [6]. Reports in the literature indicate that these metabolic changes in the heart cause alternations in both mechanical and electrophysiological properties of the myocardium [7], [8], [9]. Cardiac contractility of the heart muscles are the components of mechanical function, but contraction of the heart is ultimately regulated by electrical activity of potassium, calcium and sodium channels.

Diabetic cardiomyopathy (DCM) is a common complication of diabetes and left ventricular hypertrophy (LVH), diastolic left ventricular dysfunction, myocardial fibrosis and systolic dysfunction are some key characteristics of DCM [10]. Although a number of miRNAs have been shown to regulate variety of heart diseases including myocardial ischemia, cardiac fibrosis, cardiac arrhythmias, and heart failure [11], [12], [13], [14], the role of these miRNAs and their target genes/signaling pathways in regulating diabetic cardiomyopathy remain unknown. In the present study we utilized db/db mice as an experimental model to study the molecular mechanisms involved in electrical remodeling.

Mechanical and electrophysiological dysfunctions in heart failure are often observed with reduction of Kv4.2 expression and increased Kv1.4 expression [5], [15]. The role of Kv4.2 channel has been previously tested in the heart and brain. Although the physiological role of Kv4.2 is not entirely known, previous report [16] using the Kv4.2 knockout mouse model demonstrated that the I_to,f_ component is eliminated whereas the I_to,s_ encoded by Kv1.4 is up-regulated in the ventricles. But no changes were observed in terms of ventricular hypertrophy. This study demonstrated the essential role of Kv4.2 in generation of I_tof_ in mouse ventricle.

Another report by Barry et al. [17] showed that the ventricular action potential was prolonged and also the QT interval was prolonged at ECG level in dominant negative point mutant variant of Kv4.2 (Kv4.2DN). Therefore the authors in this study concluded that electrical remodeling occurs in the Kv4.2DN variant mouse heart.

Lugo et al. [18] examined the role of Kv4.2 in learning and memory and found that the Kv4.2KO mice are deficient in learning phase as noted by using the Morris water maze. Over all the report suggest that important role of Kv4.2 in learning and memory by using Kv4.2 KO mice. A report by Yuan et al. [19] tested the role of Kv4.2 in cortical pyramidal neurons and effects on action potential waveforms and firing in cells. Kv4.2 dominant negative variant caused action potential prolongation along with an increase in firing rates. The authors concluded that Kv4.2 plays an important role in encoding I_A_ channels and in repetitive firing in visual cortical pyramidal neurons.

Recent studies suggest the role of miRNAs in cardiovascular development and heart diseases [20]. One recent study showed that miR-1-2 regulates Kv4.2 expression, albeit indirectly through IRX5 [21]. Another report suggests that miR-301 modulates NFkB in pancreatic cancer [22], which is also known to regulate Kv4.2 expression via KChIP2 [23]. For instance, studies on miR-1 and miR-133a suggest their direct relation in cardiac arrhythmia and hypertrophy in diabetes [13], [24]. Therefore, it is conceivable that microRNA may regulate Kv4.2 expression directly. To test this, we assessed the role of miR-301 as a novel modulator of Kv4.2 in the context of diabetes.

We hypothesized that the Kv4.2 expression in diabetic heart is mediated by miR-301, which is independent of NFkB and Irx5. We utilized a multipronged approach to develop the molecular understanding Kv4.2 channel regulation in diabetes, with special focus on miR-301. For the first time, we have identified and characterized miR-301 as a novel regulator of Kv4.2 expression, and thereby contributing to electrical remodeling in diabetic heart. The observations presented in this study are therefore important for the complete understanding of disease progression and to develop targeted therapy.

## Methods

### Mice protocol

Age matched (13–14 week) mice homozygous for the diabetes spontaneous mutation (Lepr^db^) also referred as db/db and litter mate wild type (C57BL/6J) control mice (Wt) were obtained from Jackson Laboratory. All procedures employed for handling and experimentation using mice were approved by Institutional Animal Care and Use Committee (IACUC) at the University of South Florida, Tampa, USA. The mice were housed in cages with access to food and water *ad libitum*. Body weights were recorded for all mice before euthanizing with Somnasol (50 mg/kg, i.p.), hearts were collected by performing thoracotomy, and blood was also collected during this procedure. Excised hearts were rinsed in ice cold PBS (Invitrogen) and snap frozen in liquid nitrogen and immediately stored at −80°C until use. Right tibia of each mouse was also excised and length was measured for normalizing physical parameters. Whole hearts were weighed and dissected in ice cold PBS buffer containing protease and phosphate inhibitors in to distinct regions (right ventricle; RV, left ventricle; LV, septum (sep), apex and atria). Both the body weight and heart weight were normalized to tibia length.

### H&E staining and qualification

The frozen hearts were used for assessing the morphology and structure of db/db and Wt hearts. Briefly, hearts were mounted at the apex using the OCT mount (Fischer Scientific) in a cryostat (Microm HM 505N). Transverse sections of 20 micron thickness were excised from comparable regions of the Wt and db/db hearts. Sections were fixed to the glass slides by heating at 37°C for 45 minutes. After alcohol rinse and staining with hemtoxylin and eosin, the sections were washed in gradient alcohol and later dried in the humidified chamber and permanently mounted using DPX mounting solution (Fisher Scientific, CA, USA) and cover slips were placed. H & E stained heart sections were visualized under microscope (Zeiss) at 1X magnification under bright light and images were acquired using Zeiss axiocam color camera (Carl Zeiss, CA, USA). Analysis of the different regions of the heart was performed by using *ImageJ* software for volume and height measurements.

### RNA isolation and qRT-PCR

Total RNA was isolated from right ventricle or the epicardium of the left ventricular region by using Exiqon RNA isolation kit. All the procedures for the total RNA isolation along with small RNAs was followed according to manufacture protocol. The column purified and eluted RNA was quantified using a NanoDrop. 1 µg of RNA in 20 µl reaction was reverse transcribed utilizing an iScript reverse transcription kit (Bio-Rad laboratories, CA, USA). Custom gene specific primers were designed specifically for qRT-PCR using Primer-BLAST (Table 1). Gene expression in three mice of each group (*n* ***=*** *3*) was measured in triplicates in a total of 10 µl reaction using SYBR Green. All the procedures and methods are followed according to our previously published protocols [25]. All data was normalized using hypoxanthine-guanine phosphoribosyl transferase (HPRT) as housekeeping gene.

**Table 1:** Sequence of primers used for quantitative real-time PCR.

Gene	Forward	Reverse
**Kv4.2**	GCCGCAGCACCTAGTCGTT	CACCACGTCGATGATACTCATGA
**Kv1.4**	CATTTGGTTTCCCAATGGTC	GTGGTGCATTCCTTGTTCCT
**Kv2.1**	CACACAGCAATAGCGTTCAACTT	AGGCGTAGACACAGTTCGGC
**Kv4.3**	CCTAGCTCCAGCGGACAAGA	CCACTTACGTTGAGGACGATCA
**KChIP2**	GGCTGTATCACGAAGGAGGAA	CCGTCCTTGTTTCTGTCCATC
**Kv10.2**	GCAACAGGACAAGTTCTCTGGATGT	GCCAGGAGAGCACGAAGGCA
**Kv1.5**	CAGGGGCAGCAGCTTCTTGACG	CGCTGGCTCTAGGCTGGCTG
**Scn1b**	TGC TCA TTG TGG TGT TGA CC	CCT GGA CGC CTG TAC AGT TT
**Scn5a**	GGA GTA CGC CGA CAA GAT GT	ATC TCG GCA AAG CCT AAG GT
**Mef2c**	GGGGTGAGTGCATAAGAGGAG	AGAAGAAACACGGGGACTATGGG
**Gata4**	GCAGCAGCAGTGAAGAGATG	GCGATGTCTGAGTGACAGGA
**Gata6**	CTACACAAGCGACCACCTCA	CCAGAGCACACCAAGAATCC
**Irx5**	GCAAGGGCGACTCCGA	CGCAGCCGCCTTCTG
**Ezh-2**	TTACTGCTGGCACCGTCTGATGTG	TGTCTGCTTCATCCTGAGAAATAATCT
**NFkB-1**	GAGGAGTACGAGCAAATGGTGAAG	ATTGCCAAGTGCAGGAACGA
**NFkB-2**	TAAAGGCTGGTGCTGACATCCA	GGTGTTCTGAGCAGCATTTAGCAG
**MMP9**	GCGTGTCTGGAGATTCGACTTG	CATGGTCCACCTTGTTCACCTC
**MHC-α**	TACACTCTTCTCTACCTATGCTTCT	CACTATCTTCTTGAACTCAATGC
**MHC-β**	TGAATGAGCACCGGAGCAA	CTGGCTGGTGAGGTCATTGA
**Pitx2c**	GCCCACATCCTCATTCTTTC	CCTCACCCTTCTGTCACCAT
**Six1**	TTAAGAACCGGAGGCAAAGAG	CTTCTGAGCTGGACATGAGC
**Hifα**	TCTGGATGCCGGTGGTCTAG	TGCAGTGAAGCACCTTCCAC
**Gja1**	TGGGGGAAAGGCGTGAGGGA	ACCCATGTCTGGGCACCTCTCTT
**PPARγ**	GCCTGCGGAAGCCCTTTGGT	AAGCCTGGGCGGTCTCCACT
**Srf**	GCCCCACAGCAAGCGTCTCC	GGGCAACGTCACTGTCCCGC
**Dicer1**	TGCTCGAGATGGAACCAGA	TCAGCTGTTAGGAACCTGA GGC
**Drosha**	GGATAGGCTGTGGGAAAGGA	CTTCTTGATGTCTTCAG CCTCC
**Exportin-5**	CCACTTCAAACGTCTAATCGCT	GCCGGAGAAGGAT GCC

### Western Blotting

We utilized previously published protocols for Western blotting [26] with minor modifications. In brief, tissue homogenization was performed using T-PER tissue protein extraction buffer (Pierce, IL, USA) supplemented with DTT (10 mM), protease inhibitors, 1∶100 (Sigma-Aldrich, MO, USA) and phosphate inhibitors, 1∶100 (Sigma-Aldrich). The right or the total left ventricular region of the heart was minced in to small chunks using ice cold protein extraction buffer and homogenized using a hand-held homogenizer. Then the extract was centrifuged at 14000 rpm at 4°C and supernatant was collected and stored at −80°C until further use. The protein lysates were quantified by using Pierce660 buffer at 660 nm according to the manufacturer's protocol by using a microplate reader (BioTek, VT, USA). About 100 µg of protein was loaded with SDS sample buffer (Bio-Rad) and resolved using12.5% SDS-PAGE gels (BioRad). Subsequently, the proteins were transferred on to the PVDF membrane for immune detection of candidate proteins. Antibodies were obtained for Kv4.2 (1∶1000), Kv1.4 (1∶100), NFκB-p105/50 (1∶1000) and GAPDH (1∶10000) from Millipore (Billerica, MA, USA), KChIP2 (1∶1000), Irx5 (1∶100), myosin heavy chain-α/MHC-α (1∶200) and myosin heavy chain-β/MHC-β (1∶10) from Abcam (Cambridge, MA, USA). Secondary antibodies were either rabbit anti-mouse or goat anti-rabbit at a dilution of 1∶10,000 (Millipore, MA, USA). The blots were developed by and exposed on to the X-ray films (Santa Cruz Biotechnology). Bands were analyzed using *ImageJ* software for quantification, and normalization was done using GAPDH band intensities.

### ELISA

The blood was collected from the mice after euthanasia by using sterile syringes and transferred in to vials containing anticoagulant and centrifuged at 250 rpm at 4°C for 10 minutes to separate the plasma from blood cells. The plasma was transferred to fresh vials for measurement of extracellular TNFα by using ELISA Kit (Invitrogen, NY, USA). For intracellular TNFα measurements, the LV and RV tissues were homogenized according to the manufacturer protocol. The tissue homogenate or the plasma was used on the ELISA plate and probed for measurement of TNFα levels. The levels were quantified by extrapolating to the standard curve and plotted as bar graphs. All the procedures for TNFα ELISA were followed according to manufactures protocol and all the values are represented in fold with mean (±SEM, n = 5).

### Cell culture (H9C2) and transfection procedures

The H9C2 cells were purchased from ATCC (Manassas, VA, USA). H9C2 cells are rat cardiomyoblasts and were cultured in 5% CO_2_ incubator (Thermo Fischer Scientific, IL, USA) using standard DMEM medium (Invitrogen) supplemented with 10% Fetal Bovine Serum (Invitrogen), penicillin and streptomycin antibiotics. For miRNA transfection experiments, the cells were transfected with 50 nM of either scrambled inhibitor or miR-301 inhibitor (Cat# 4464084, Applied Biosystems, CA, USA) at 70% confluence using Lipofectamine™ LTX transfection system (Invitrogen). Cells were observed for any toxicity for every 24 h under microscope. No detectable cell loss or change in cell morphology was observed in inhibitor treated group. Total RNA including small RNAs were extracted after 24 h, 48 h and 72 h of post-transfection using Exiqon RNA isolation kit as described above.

### TaqMan PCR

The miR-301 expression was measured by using TaqMan miRNA assay kit (Applied Biosystems) using total RNA including small RNAs isolated from LV (epicardium) and RV as described above. A total of 100 ng of RNA was reverse transcribed using TaqMan miRNA reverse transcription kit (Applied Biosystems) using specific primers for miR-301a and U6. TaqMan primers specific to miR-301a and U6 were obtained from Applied Biosystems and quantification was done using universal TaqMan master mix (Applied Biosystems) as per the manufactures protocol. Similar procedures were also followed for the estimation of miR-301a expressions in the cells treated with miR-301a inhibitor. All the procedures, cycle conditions and analysis methods were followed according to our standard protocols [27]. All the values (mean ± SEM, n = 3–6) were presented as fold expressions after normalizing with the endogenous control U6.

### MicroRNA-gene targeting experiment

Estimation of putative target for miR-301a was done using various *in silico* software including miRDB.org (http://mirdb.org/miRDB/) and miRanda (http://www.microrna.org/microrna/home.do). The putative binding site of miR-301a on mouse Kv4.2 gene was also identified by using miRanda software and the ‘seed’ sequence from the 3′-untranslated region (3′-UTR) of Kv4.2 was synthesized (IDT, NJ, USA). Sense and anti-sense oligo-nucleotide sequences (miR 301a-‘seed’ sequence) synthesized were annealed and ligated to pmiRGLO Dual-Luciferase miRNA Target Expression Vector system (Promega, WI, USA). Positive clones were confirmed by DNA sequencing at Moffitt DNA Core facility (Tampa, FL, USA). Endotoxin free plasmid from the positive clones carrying miR-301a binding site on 3′UTR of Kv4.2 was isolated using Qiagen plasmid midi extraction kit (Qiagen, CA, USA). The rat cardiomyocyte cells (H9C2) were transfected with either pmiRGLO miR-301a target sequence plasmid or positive control plasmid (pGL3) with similar background without target sequence at 80% confluence using Lipofectamine™ LTX (Invitrogen) according to the manufactured protocol. After 24 h of post transfection, the cells were added with 50 nM of either scrambled inhibitor (Cat# 4464076), or miR-301a inhibitor (Cat# 4464084) or miR-301a mimic (Cat# 4464066) and incubated in CO_2_ incubator for another 48 h. The cells were then lysed with 1X passive lysis buffer and luciferase activity was measured using Dual-Luciferase assay kit (Promega). *Renilla* expression plasmid (pRL-TK vector) from Promega was co-transfected with pmirGLO or positive control (pGL3) as an internal control and all the values were normalized with *Renilla* counts and normalized RLUs were expressed (mean ± SEM, n = 6).

### Patch-clamp recording

Whole cell patch-clamp recordings were performed on H9C2 cells transfected with scrambled or miR-301a inhibitor using protocols similar to those we have previously published [26], [28]. Recordings were made from cells plated on glass coverslips and after 72 hours of treatment with the control (scrambled) or miR-301a inhibitor. The external solution for recordings consisted of (in mM): NaCl 135, KCl 5.4, MgCl_2_ 1.0, CaCl_2_ 1.8, HEPES 10 and Glucose 5.5 at pH 7.4. We utilized Axopach 200B amplifier and Clampex 10 software to perform the voltage-clamp acquisition and analysis (Clampfit). The internal (patch pipette) solution consisted of (in mM): Aspartic acid 100, KCl 35, MgCl_2_ 1.0, CaCl_2_ 1.8, NaCl 4.5, EGTA 10, ATP 5 at pH 7.2 with KOH. Patch pipettes with 1–3 MΩ resistance were used to obtain GΩ tight seals and membrane under the patch pipette was ruptured using negative pressure to achieve the whole-cell configuration. Fast inactivation was determine by applying depolarizing pulses from −20 to +60 mV in 10 mV steps to the cells from a holding of −80 mV for 500 ms, followed by a step to −10 mV. Analysis of the current decay was subsequently performed by calculating tau (τ) using a monoexponential equation from I_peak_.

### Pathway and Network Analysis

Differentially expressed genes with p-value≤0.05 from qRT-PCR data were selected for network analysis using Ingenuity Pathway Analysis (IPA) software (Ingenuity Systems, Inc., CA, USA). Based on the existing literature, IPA identified the networks from its library of canonical pathways that were most significant to the data set. The significance of the association between the data set and the pathway network was measured by a ratio of the number of genes from the data set that map to the pathway divided by the total number of genes that map to the canonical pathway is displayed.

### Statistical analysis

Data were obtained from 3–6 mice of each group in duplicates or as indicated. The qRT-PCR data was obtained in triplicates. All data analyzed in this study are expressed as mean± SEM. A two-tailed, student t-test was utilized to detect variables that differed significantly between the db/db and Wt groups. Only the values with p≤0.05 were considered as statistically significant for all mean comparisons between the two groups.

## Results

### Structural remodeling of diabetic heart

**Figure 1:**
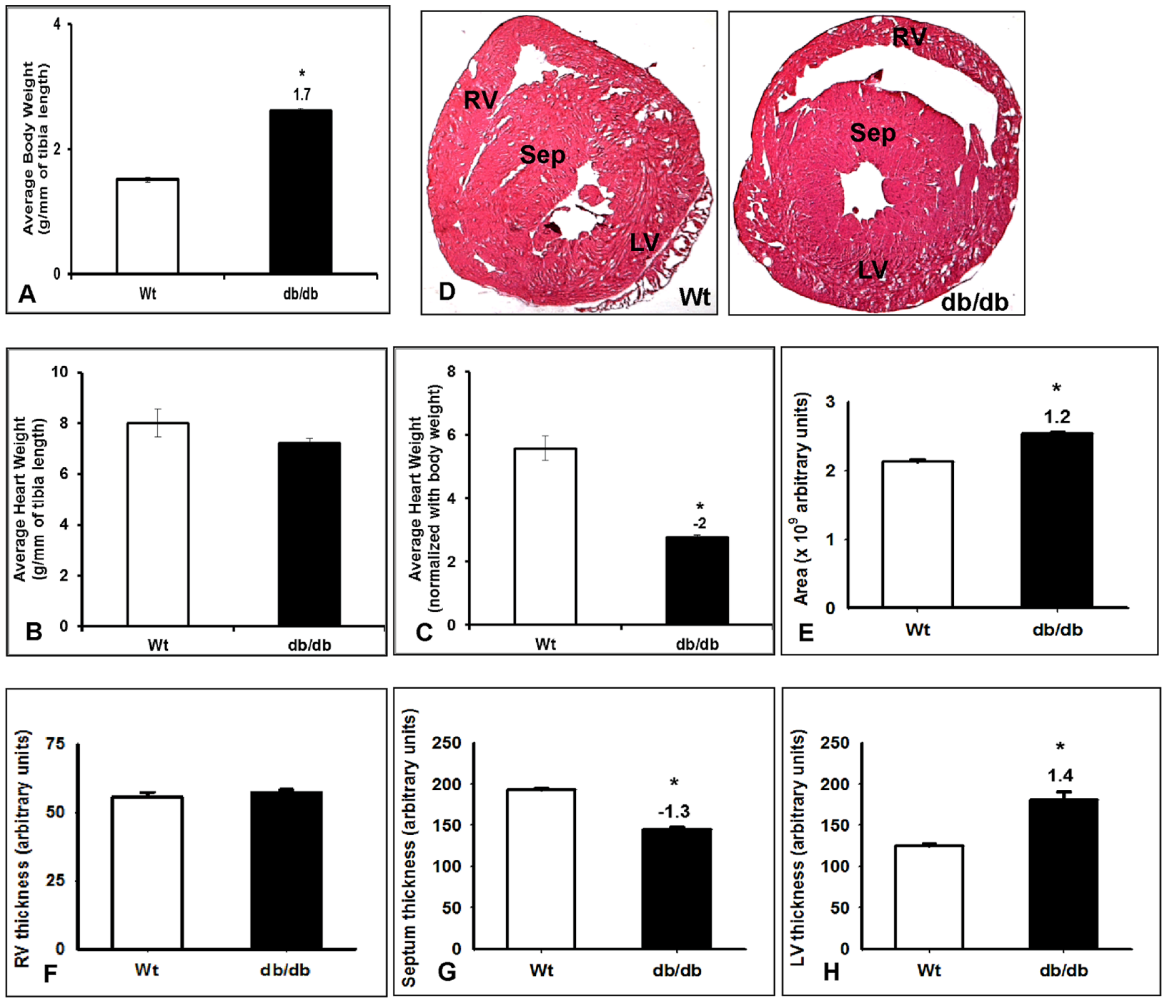
Physical parameters of the db/db and age matched wild type (Wt) mice. The averaged body weights of db/db and Wt group normalized to tibia (A), heart weights normalized to tibia (B) and body weight(C), H&E staining of db/db and Wt hearts (D), cross-sectional area of the heart (E), RV thickness (F), Septal wall thickness (G) and LV thickness (H) are presented. All values presented in the bar diagram are mean (±SEM n = 15–16) with *p≤0.05.

Age-matched (13–14 week) db/db mice and Wild type (Wt) mice from Jackson Laboratory were used in the study. We observed a significant increase in body weight of db/db group (n = 15) compared with wild type (n = 16) controls (Fig.1a). Body weight and heart weight of each mouse was normalized to their respective tibial length. As shown in Fig 1b, we did not observe significant change in normalized heart weights of db/db group compared with wild type controls. However, when heart weight was normalized to body weight, we found a significant decrease in the db/db group compared to wild type (Fig.1c). Morphometric analysis using H&E staining showed a significant increase in cross-sectional area of db/db hearts compared to wild type group (Fig.1e). Although there is no significant difference in the RV wall thickness, we observed a significant increase in LV wall thickness, and a decrease in septal wall thickness in db/db hearts compared with wild type (Fig.1f–h).

### Differential expression of genes involved in right ventricular remodeling in diabetic heart

**Figure 2:**
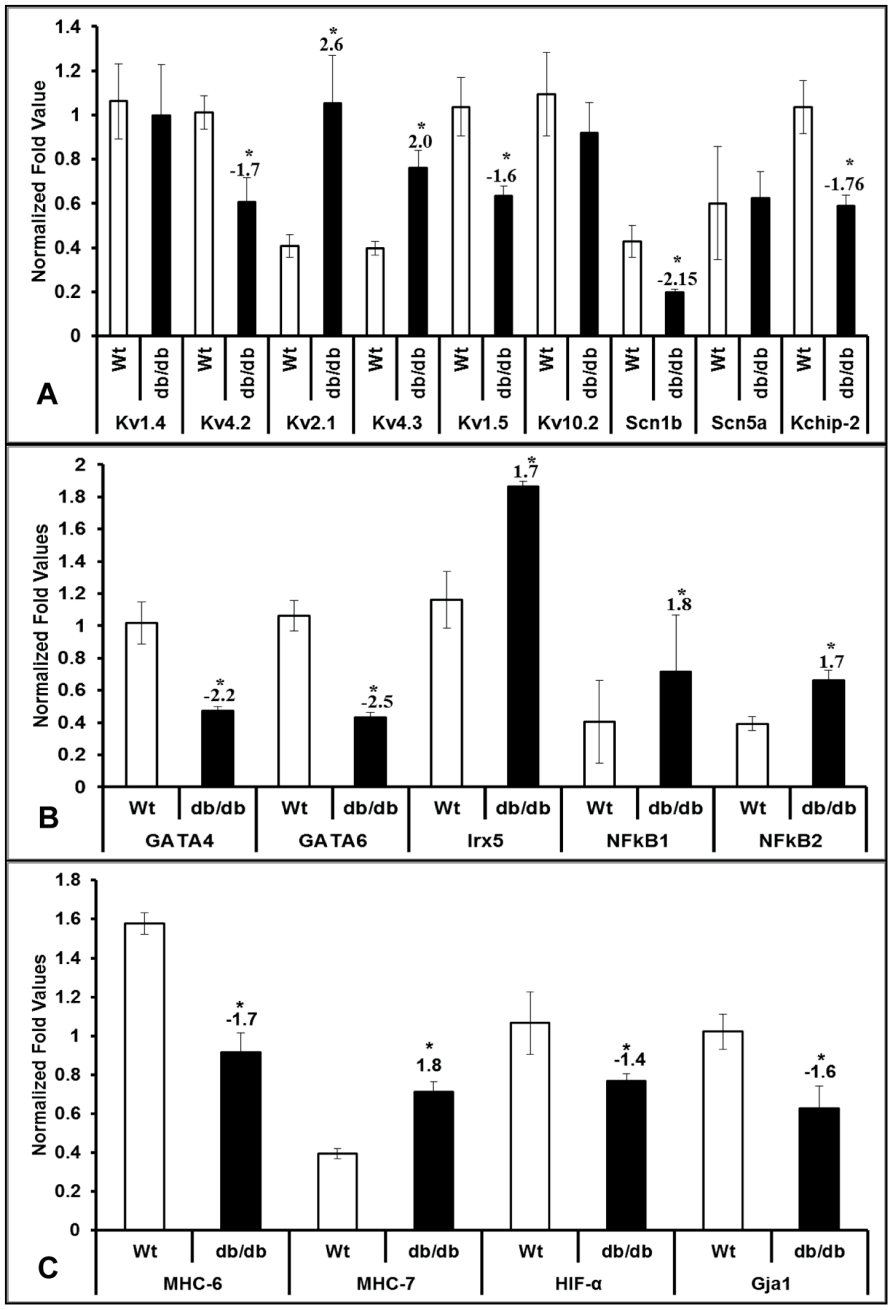
Transcriptional regulation in the right ventricle of diabetic (db/db) heart. Quantitative real-time PCR (qRT-PCR) in right ventricle (RV) of wild type (Wt) and diabetic (db/db) mouse hearts with potassium channels Kv1.4, 4.2, 2.1, 4.3, 1.5, 10.2, and sodium channels Scn1b and Scn5a, along with Kv channel gene chaperon KChIP2 (A), transcriptional factors such as GATA4, GATA6, Irx5, NFkB (B), and Hif1α, along with MHC-α, MHC-β and Gja1 (C). Bars represent mean (±SEM) expression in fold, n = 3 and * represents p≤0.05.

Previous reports show systolic and diastolic dysfunctions in 12 wk old db/db mice [29], therefore we investigated the expression of ion channel genes, chaperons, transcriptional factors and hypertrophic markers in both right and left ventricles of db/db hearts using quantitative real-time PCR (qRT-PCR). Out of 30 genes tested using qRT-PCR, we observed a significant change in expression of 17 genes in RV and 12 genes in LV of db/db mice compared with wild type controls. Among the ion channel genes, Kv4.2, Kv1.5, and Scn1b are significantly down-regulated in right ventricle; whereas Kv2.1 and Kv4.3 were significantly up-regulated (Fig.2a). There is no significant change in Kv1.4, Kv10.2, and Scn5a expressions in RV of db/db hearts compared with wild type controls. As Kv4.2 transcripts in the RV were significantly decreased, we evaluated the expression of potassium channel interacting protein; KChIP2 and observed a significant reduction (Fig.2a). We also observed a significant increase in the expression of Kv4.2 repressor, Irx5, in right ventricle of db/db hearts (Fig.2b).

Other transcriptional factors that are specific to heart such as GATA4, GATA6, Mef2c, Pitx2c, and Hif1α, were significantly down-regulated in RV compared to wild type controls (Fig.2 b and c, Table.2), while NFκB1 and 2 were significantly up-regulated (Fig.2b).

In addition to these transcriptional factors, hypertrophic markers such as MHCα & MHCβ, metalloprotease MMP9, and miRNA processing enzymes such as Dicer, Drosha and Exportin5 were also examined in RV of db/db hearts. Our data showed that there is an isoform switch in MHCs expression where there is reduction of MHCα and increase in MHCβ transcripts (Fig.2c). We observed a significant reduction of Gja1 (Cx43) and MMP9 in RV of db/db mice compared with wild type controls (Fig.2c and Table.2). Other genes such as Ezh-2, Six-1, PPARγ, Srf, Dicer, Drosha and Exportin5 were not changed significantly in RV of db/db hearts compared to wild type (Table.2).

**Table 2:** Quantitative real-time PCR of differentially expressed genes in right ventricle of diabetic mice.

Gene	Average Fold	SEM (n = 3)	p-value
**Mef2c**	−2.8	0.027	0.003
**Ezh-2**	1.27	0.05	0.11
**Six-1** ****	−1.11	0.09	0.42
**Pitx2c**	−2.7	0.13	0.04
**MMP9**	−2.3	0.035	0.002
**PPARγ**	1.07	0.072	0.34
**SRF**	−1.14	0.1	0.185
**Dicer**	−1.03	0.06	0.31
**Drosha**	1.02	0.03	0.33
**Exportin-5**	−1.3	0.03	0.07

### Altered expression of genes involved in left ventricular remodeling

**Figure 3:**
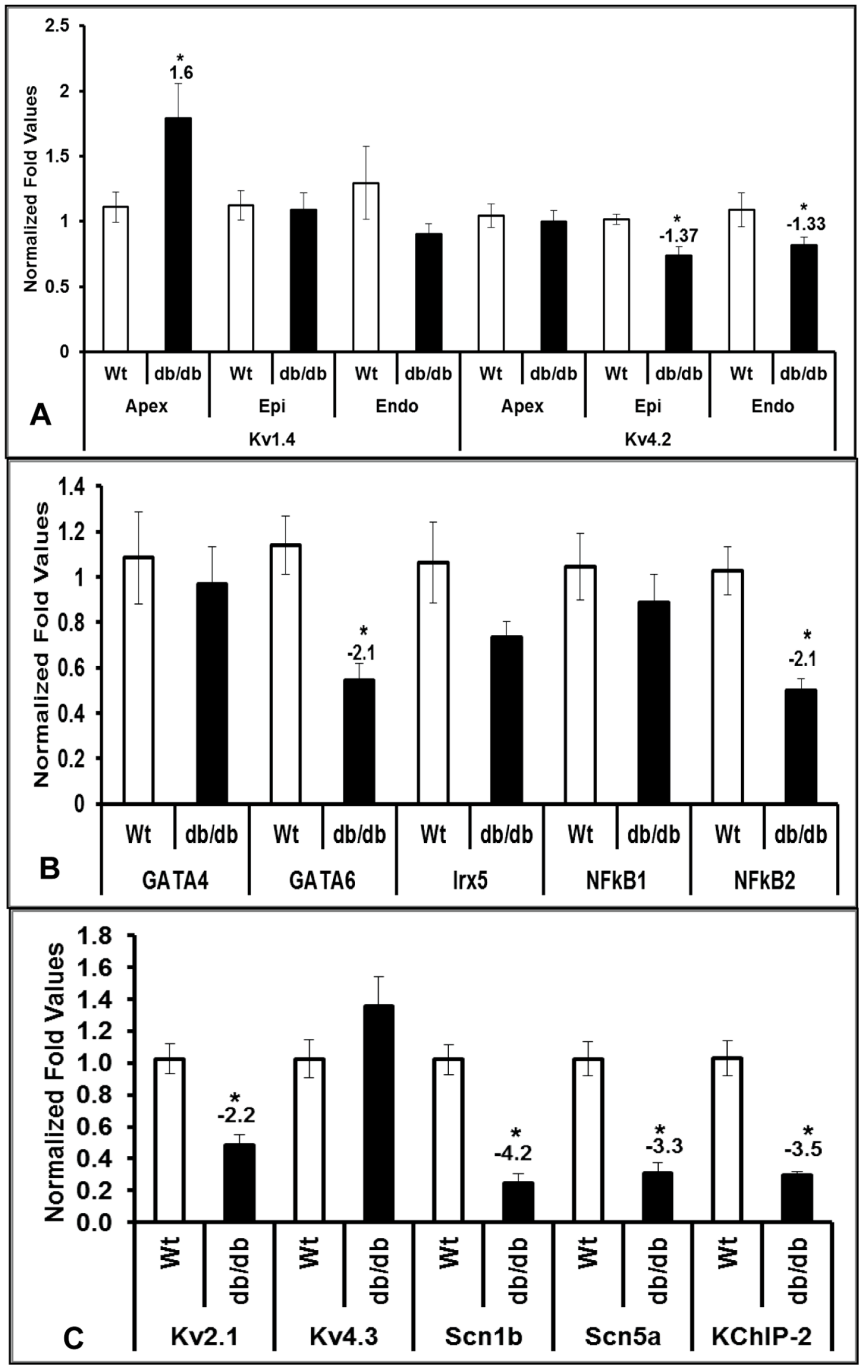
Differential expression of genes in left ventricle. The qRT-PCR analysis of Kv4.2 and 1.4 in epicardium, endocardium and apex region (A), GATA4, GATA6, Irx5, NFkB (B), Kv2.1, Kv4.3, Scn1b, Scn5a, and KChIP2 (C) in epicardium of left ventricle (LV) from wild type (Wt) and diabetic (db/db) mouse hearts. Normalized fold values were expressed in bar diagrams are mean (±SEM) of n = 3 and * represents p≤0.05.

Since cross-sections of db/db hearts in the current study showed a significant increase in LV wall thickness and decrease in septal wall thickness, we anticipated differential expression of ion channels, transcriptional factors and other markers in LV also. Due to the transmural gradient in expression of Kv4.2 and Kv1.4 genes in LV, we utilized the epicardium and endocardium and examined the gene expression profiles separately along with apex region where the expression of Kv4.2 and Kv1.4 is homogenous. Our data showed a significant reduction of Kv4.2 in both the epicardium and endocardium of the LV but no change in apex of db/db hearts when compared to wild type controls (Fig.3a). However, we did not see any significant alteration in Kv1.4 gene expression in epicardium and endocardium of the LV, whereas a significant increase in apex was observed (Fig.3a). Similar to RV, we also observed a significant reduction of GATA6, KChIP2, and Scn1b in epicardium of LV. However, in contrast to RV, we observed increase in expressions of Mef2c and reduction of NFkB2, Kv2.1 and Kv4.3 expressions in LV (Fig. 3b&c and Table.3). In addition to these changes, we also observed a significant reduction of Ezh2 and Six1 in LV epicardium in db/db mice compared to wild type controls (Table.3). We did not observe any significant change in MHCβ, BMP10, Pitx2c (Table.3), GATA4, Irx5, and NFkB1 (Fig.3b&c) in LV of db/db hearts.

**Table 3:** Quantitative real-time PCR of differentially expressed genes in left ventricle of diabetic mice.

Gene	Average Fold	SEM (n = 3)	p-value
**MHCβ**	1.04	0.11	0.42
**Mef2c**	4.8	.88	0.004
**Ezh-2**	−2.4	0.03	0.0026
**Six-1**	−2.6	0.09	0.036
**BMP10**	1.27	0.17	0.25
**Pitx2c**	1.45	0.47	0.24

### Protein expression

**Figure 4:**
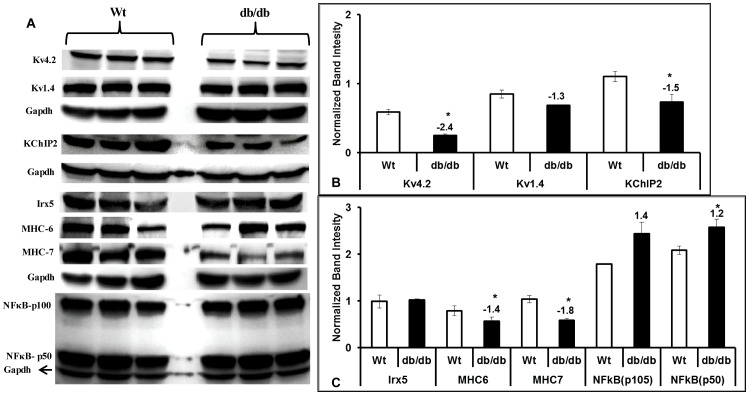
Protein profile in right ventricle of diabetic hearts. Comparative Western blot analysis of the key potassium channels along with transcription factors, chaperons and hypertrophic markers are shown (A). Band intensities for Kv4.2, 1.4, KChIP2 (B), Irx5, NFkB, MHC6 and MHC7 (C) were measured and presented as bar diagrams after normalizing with GAPDH band intensities. All the values presented here are mean (±SEM) of n = 5–6, and * represents p≤0.05.

**Figure 5:**
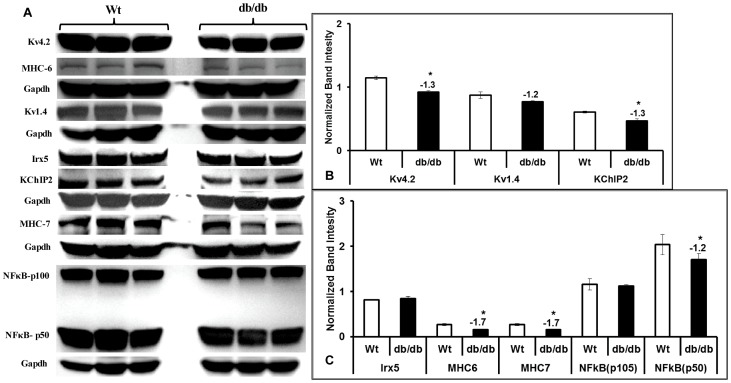
Western analysis of ion channel expression and key proteins. Protein profiling of differentially expressed genes in left ventricle of db/db group compared with wild type (Wt) controls (A). Band intensities for Kv4.2, 1.4, KChIP2 (B), Irx5, NFkB, MHC6 and MHC7 (C) were measured and presented as bar diagrams after normalizing with GAPDH band intensities. All the values presented here are mean (±SEM) of n = 5–6, and * represents p≤0.05.

Based on the changes observed at mRNA levels in RV and LV we identified key proteins for evaluation by Western blotting. Western analysis in RV of db/db heart showed a significant reduction of Kv4.2, KChIP2, MHCα and an increase in NFkB (Fig.4), which is consistent with qRT-PCR data. However, we observed down regulation of MHCβ and no significant change in Irx5 in RV of db/db hearts compared with wild type controls (Fig.4). Similarly, we observed directional correspondence with qRT-PCR data for Kv4.2, KChIP2, and NFkB protein expression data in LV of db/db hearts, but no significant change in Irx5 and Kv1.4 protein expressions (Fig.5). Similar to RV, we also observed a significant reduction of both MHCα and MHCβ proteins in LV of db/db hearts compared with wild type controls (Fig.5).

**Figure 6:**
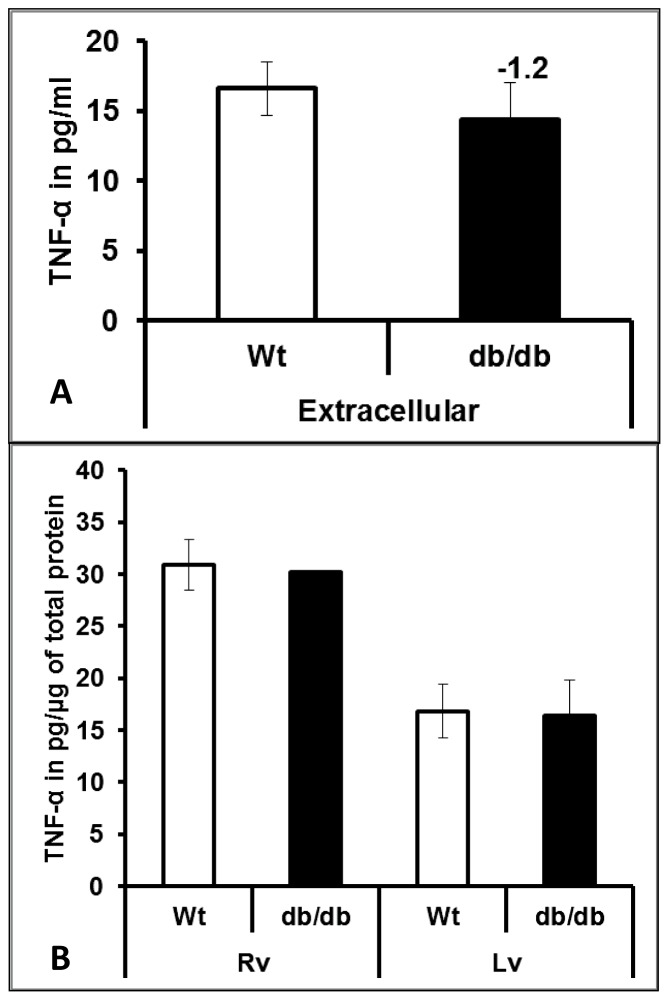
Extracellular and intracellular expressions of TNF-α in diabetic hearts. Plasma and tissue homogenates were used for measurement of TNF-α by using ELISA kit. The right and the left ventricle were evaluated separately for measuring the TNF-α in a regional specific manner. The values are presented as bar diagrams of either TNFα protein expression in pg/ml of plasma (A) or pg of TNFα/µg of total protein (B). All the values presented here are mean (±SEM) of n = 5.

Since there is significant increase in expression of NFkB both at the mRNA and protein level in RV of db/db mice, and TNF-α is known to induce NFkB expressions [30] and also regulate Kv4.2 expression [31], [32], [33], we investigated if this increase is due to the induction of cytokine TNF-α. ELISA results from our study showed that there is no significant change in TNF-α expression both at plasma as well as intracellular (LV and RV) in db/db mice compared with wild type controls (Fig.6).

### MicroRNA-301a directly regulates Kv4.2 gene expression in diabetic hearts

**Figure 7:**
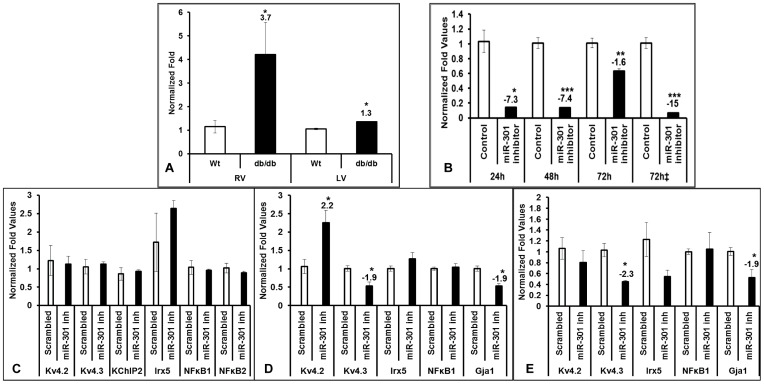
MicroRNA-301a regulated expression of Kv4.2. The expression of miR-301 in db/db and Wt mouse right and left ventricles were assessed by Taq-man PCR (A). Rat cardiomyocytes (H9C2) were transfected with either scrambled or miR-301 inhibitor (50 nM) for different time points 24, 48 and 72 hours and miR-301a expressions were quantified using TaqMan assay (B). The bars labeled with 72‡ are the cells transfected with inhibitor (50 nM) for 72 h with additional supply of inhibitor at 48 h. The expression of Kv4.2, Kv4.3 KChIP2, Irx5, and NFkB were quantified using qRT-PCR for the cells transfected with miR-301a inhibitor or scrambled inhibitor for 24 h (C), 48 h (D) and 72 h (E). All values are normalized with housekeeping gene (U6 RNA for TaqMan and HPRT for qRT-PCR) and plotted as mean (±SEM) of n = 3–6. * represents p≤0.05, ** with p≤0.005, and *** with p≤0.0005.

Quantitative real-time PCR and Western blotting experiments showed that Kv4.2, and its interacting protein KChIP2 and transcriptional regulator NFkB, are differentially expressed in both RV and LV of db/db hearts. Therefore, we examined if these changes are due to microRNAs. For this, we investigated the expression profile of miR-301a, which is known to be a targeting miR of NFkB repressing factor (Nkrf) [22], using a TaqMan assay. Our data showed that there is a significant increase in expression of miR-301a in both RV and LV of db/db hearts compared with wild type (Fig.7a). This precipitated us to investigate if the change in expression of Kv4.2, KChIP2 and NFkB are differentially regulated by miRNA. We used loss-of-function approach in the *in vitro* model of rat cardiomyoblasts (H9C2 cells) using miR-301a inhibitors. We optimized experimental conditions by using the time course approach and identified a significant reduction in miR-301a expressions in cells treated with 50 nM miR-301a inhibitor for 24 h and 48 h (−7.3 and −7.4 fold respectively), but only −1.6 fold reduction was observed after 72 h of treatment (Fig.7b). When the cells were supplemented with additional miR-301a inhibitor after 48 h, reduction much higher reduction (−15 fold) of miR-301a was obtained (Fig.7b) suggesting that miR-301a was sensitive to specific inhibitor treatment. Then we further investigated the expression levels of Kv4.2, KChIP2, Irx5 and NFkB along with other genes in miR-301a inhibitor treated cells. Our data showed that there is no significant change in expressions of Kv4.2, 4.3, KChIP2, Irx5, and NFkB after 24 h of inhibitor treatment but significant increase in Kv4.2 expressions after 48 h of treatment (Fig.7c and d), and significant reduction of Kv4.3 and Gja1 after 48 h and 72 h treatment (Fig. 7d&e). But we did not see any significant change in expressions of Irx5, KChIP2 and NFkB at all three time periods (24, 48 and 72 h) after miR-301a inhibitor treatment (Fig.7).

**Figure 8:**
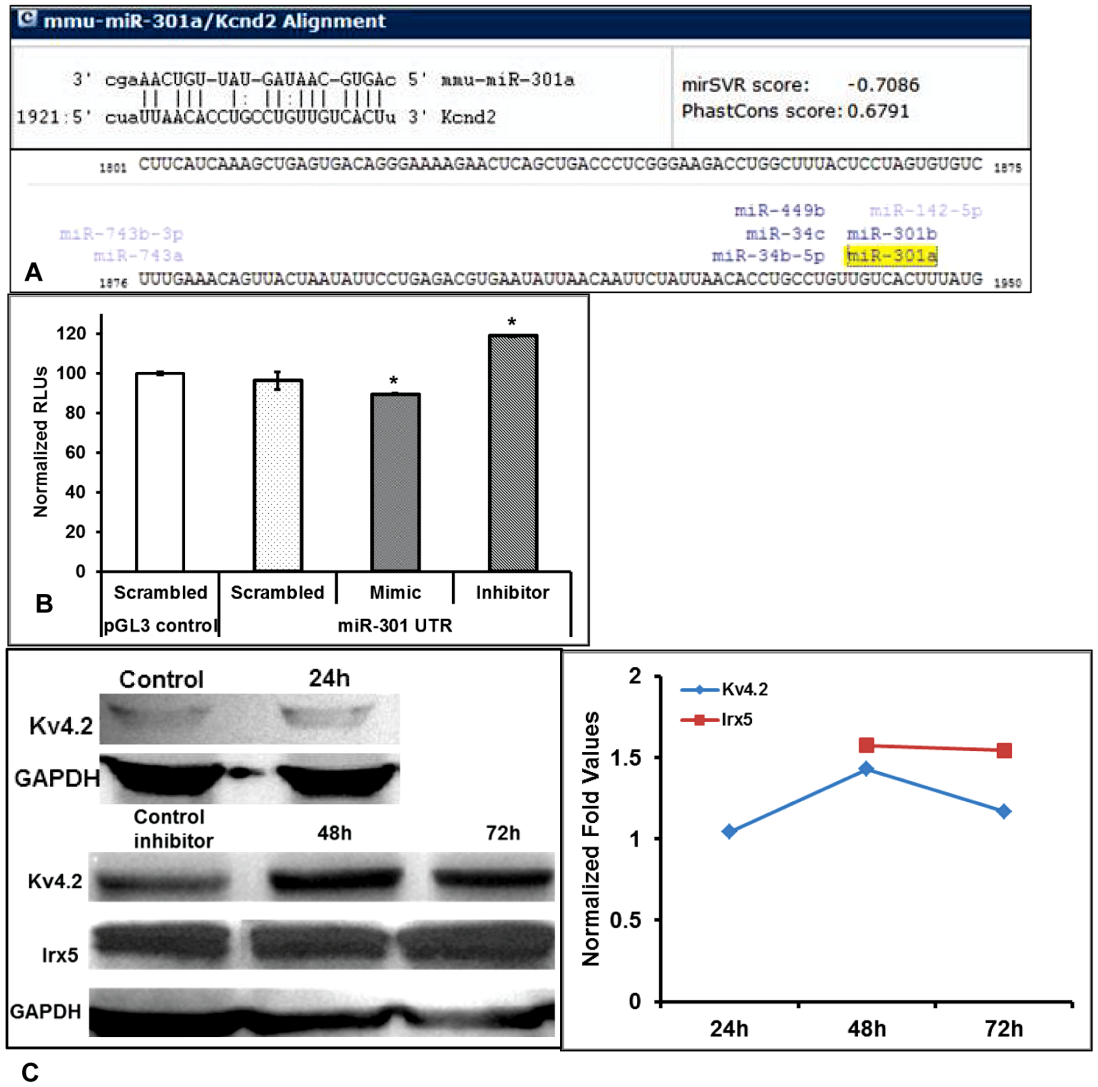
Post-transcriptional regulation of Kv4.2 by miR-301a by direct binding. The *in silico* analysis showing the direct binding of miR-301a to the ‘seed’ sequence on 3′-UTR region of Kv4.2 gene (A). Quantification of luciferase activity in H9C2 cells showing direct binding of miR-301a to the ‘seed’ sequence of Kv4.2 gene (B). Protein expression profile of Kv4.2 (24, 48, and 72 h) and Irx5 (48 h and 72 h) genes in H9C2 cells transfected with miR-301a inhibitor (50 nM) using Western blotting analysis (C). All the values presented in the bar diagram are mean (±SEM) of n = 3–6 and * represents p≤0.05.

The elevation of Kv4.2 expression observed upon miR-301a inhibitor application in the absence of changes in KChIP2, Irx5 and NFkB expression raises the possibility that miR-301a regulates Kv4.2 gene expression directly at the transcriptional level. To examine this, we initially explored *in silico* tools to predict the possible binding sites of miR-301a in 3′-untranslated regions (3′-UTR) of Kv4.2 gene. Using miRanda software, we identified miR-301a potential binding sited on 3′UTR of mouse Kv4.2 gene (Fig.8a). To further confirm our *in silico* predictions, we utilized pmirGLO-Luciferase miRNA target expression system (Promega) with the predicted miRNA-301a ‘seed’ sequence from Kv4.2 gene. We expressed this miRNA target expression system in H9C2 cells, and transfected with either 1) scrambled miRNA inhibitor sequence, or 2) miR-301a inhibitor, or 3) miR-301a mimic for 48 h. Luciferase expression was then quantified by Dual-Luciferase kit (Promega). Results from loss of function experiments utilizing luciferase assay show significant increase in luciferase activity in the cells treated with miR-301a inhibitor compared with the scrambled inhibitor (Fig.8b), whereas gain of function experiments using the cells transfected with miR-301a mimic showed a significant reduction of luciferase activity compared with scrambled group (Fig.8b). Western blotting of cells treated with miR-301a inhibitor for 48 h and 72 h further confirm these results as shown by significant increase in the expression of Kv4.2 protein without any change in Irx5 expression (Fig.8c). Together these observations suggest that miR-301a can potentially target Kv4.2 gene and directly regulate its expression.

**Figure 9:**
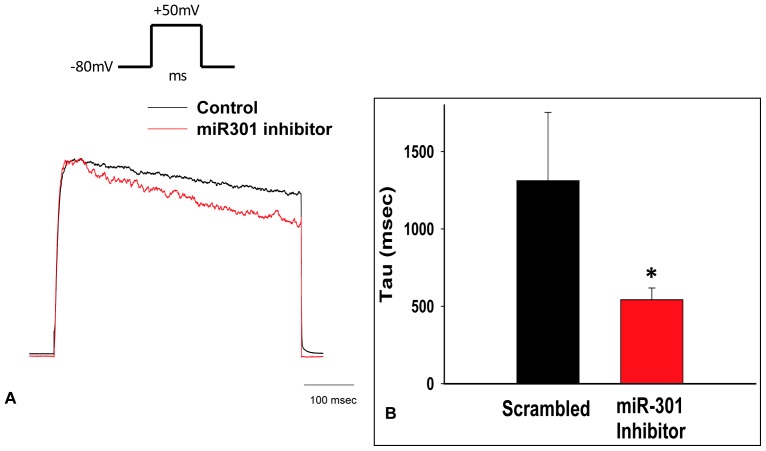
Kv current recording in H9C2 cells treated with miR-301a inhibitor. H9C2 cells were either transfected with miR-301a inhibitor (50 nM) or scrambled inhibitor and potassium channel currents were measured by whole cell patch clamp technique after 72 h of treatment. (A) Representative whole cell currents recorded from H9C2 cells normalized to I_peak_ are shown at +50 mV. (B) The tau (τ) calculated from the recordings with scrambled or miR-301a inhibitor is shown as bar graph representing mean ± SEM with p≤0.05, n = 11 for scrambled and n = 22 for miR-301a inhibitor.

We performed whole cell patch clamp experiments on H9C2 cells treated with either miR-301a inhibitor or scrambled inhibitor for 72 h. We observed a significant inhibition of miR-301 expression by 15 fold (Fig 7b). Representative Kv current traces recorded from two H9C2 cells in response to depolarizing voltage step to +50 mV are shown in Figure 9a. To facilitate comparison, currents were normalized to respective peak amplitude. Data obtained from these experiments show that the outward currents (Kv currents) obtained from H9C2 cells were significantly altered, such that the inactivating τ was faster in the miR-301a inhibitor treated group (543 ms) compared with scrambled group (>1000 ms) suggesting the modulation of Kv channel expression by miR-301a inhibitor (Fig 9b).

## Discussion

The major findings in the present study are: (1) direct regulation of Kv4.2 in diabetic heart by miR-301, (2) involvement of NFkB and KChIP2 mediation in modulating the repolarization reserve, (3) Irx5 independent modulation of Kv4.2 and (4) remodeling of right and left ventricle in db/db hearts through distinct signaling pathways. To evaluate the possible roles of novel miR-301 mediated regulation in Kv4.2 we probed by using loss-of-function and gain-of-function approach using H9C2 cells as a model and identified the distinct cellular pathway regulating the direct binding of miR-301 to Kv4.2 3′-UTR region suggesting for the first time the role of a novel miR-301 mediated regulation of Kv4.2 (Fig. 8). These observations in the *in vitro* model are consistent with the results obtained in the diabetic (db/db mice) ventricles where a significant increase in the miR-301 and significant reduction of Kv4.2 expression leading to depletion of repolarization reserve in the diabetic hearts was observed (Fig. 2–5 & 7). Together we identify the role of miR-301 as a novel repressor of Kv4.2 channel and other transcriptional factors of pathophysiological significance in diabetes.

The functional role of down regulated potassium channels in db/db mouse ventricular cardiomyocytes is reported previously by Shimoni et al. [34]. Action potentials recorded from isolated cardiomyocytes show a clear prolongation of action potential duration suggesting the direct role of depleted repolarization reserve. Earlier investigators used STZ models of diabetes and identified that the prolongation of action potential is reversed by additional supplementation of insulin in the perfusion medium during action potential recording. However, the molecular insights were lacking in db/db model for its relation to how the Kv4.2 depletion occurs. Based on the existing literature, it is suggested that Irx5 and KChIP2 are major regulators of Kv4.2 activity [23], [35], [36], [37]. Therefore, we investigated the link between K4.2, KChIP2 and Irx5 in the present study and found that KChIP2 is significantly down regulated in the right and the left ventricle (Fig.4 & 5). This is consistent with the idea that KChIP2 is a major regulator of Kv4.2 expression. Shimoni et al. [38] in another study identified that the cardiac insulin receptor knockout mice have prolonged action potentials and is not attenuated by supplementing with quinapril (angiotensin converting enzyme inhibitor) suggesting the role of insulin receptor in modulating the repolarization caused by potassium channels in action potential duration. Among the KChIP isoforms (1–3), KChIP2 is the most abundant and highly expressed in heart tissue and decreased levels of this chaperon protein has been previously reported in hypertrophy and heart failure [35], [39]. Except the expressions of KChIP2 and other transcription factors, molecular events up-stream of Kv4.2 and its regulation are not yet reported. Therefore we undertook the present study to identify the molecular mechanisms modulating Kv4.2. We identified that KChIP2 is significantly down regulated both in the right and the left ventricle suggesting an important role for potassium channel interacting protein in regulation of Kv4.2 expression. Cardiac hypertrophy and failure are associated with decreased Kv4.2 and 4.3 expressions levels [40], [41], and Kv channel-interacting proteins (KChIP) are known to interact with these Kv channels [42], [43]. Therefore we hypothesized that KChIP2 regulates Kv4.2 in this model and expected a decrease in the KChIP2 expression. We observed a significant reduction in KChIP2 at protein expression which is consistent in LV and RV region (Fig. 4 & 5). However, no differences were found in Irx5 expression in the right and the left ventricle, clearly identifying that at least in the db/db model of diabetes, Irx5 is not a major regulator of Kv4.2.

The existing reports suggests that the isoform switch between MHC-α to β in diabetic cardiomyopathy [5] with no ventricular hypertrophy. In the present investigation, we observed similar isoform switch between MHC-α to β in diabetic group compared to wild type in right ventricle using qRT-PCR (Fig.2c), but not in epicardium of left ventricle (Table.3). To evaluate the physical changes in the diabetic hearts we performed morphological measurements (Fig.1). In consistent with the previous report [5] we did not find major changes in average weights of the db/db hearts compared with Wt after normalizing with their tibia lengths (Fig.1c), but, observed a significant reduction in heart weights when normalized with body weights (Fig.1d). Moreover, we also found a significant change in the average cross-sectional area of the heart which is contributed from the overall change in the septal and left ventricular free wall area (Fig 1g&h). Earlier reports have [44] noted functional changes in the db/db hearts using echocardiographic measurements where the left ventricular diastolic and systolic dimensions were significantly increased along with a decrease in fractional shortening suggesting overall changes in the diabetic hearts at muscle. Another report by Yue et al. [45] identified larger hearts in the 17 week old db/db mice compared with Wt by using magnetic resonance imaging. Structural and functional parameters as noted by posterior wall thickness, anteroseptal wall thickness, left ventricular ejection fraction and end diastolic volume were also significantly different at the 17 week time point in the db/db hearts suggesting a pattern consistent with ongoing remodeling in the db/db mouse hearts. Our results are consistent with previous reports revealing the physical measurements and overall changes in the db/db heart. This further support the observations by other investigators that although there is no hypertrophy in diabetic heart, isoform switch in cardiac myosin heavy chains still exist. Majority of cardiomyopathies are characterized by electric remodeling [5], [32], [36], leading to left ventricular hypertrophy and heart failure. The present study shows ion channel remodeling which may lead to cardiac failure in db/db mice. Cardiac contractility of the heart muscles are the components of mechanical function, which are attributed by electrical activity of potassium, calcium and sodium channels. Mechanical and electrophysiological dysfunctions in heart failure are often observed with reduction of Kv4.2 expressions and increased Kv1.4 expressions [5], [15]. A study by Qin et al. [15] showed that streptozotocin (STZ) injected type I diabetic rats showed a significant reduction of Kv4.2, 4.3 and 2.1 transcript as well as protein levels in left ventricle. In another study by Nishiyama et al. [5], the STZ-induced diabetic rats showed a significant reduction of Kv4.2 transcripts as well as protein and increased Kv1.4 transcripts and protein levels, but no significant change in Kv4.3 expressions in ventricle. Our data showed a significant reduction in Kv4.2, but no significant change in Kv1.4 in both RV and LV (Fig. 2, 3, 4 and 5). In contrast to previous studies, we found a significant increase in transcripts of Kv2.1 and 4.3 levels in RV (Fig.2). But, epicardium of LV showed significant reduction of Kv2.1 transcripts which is in correspondence with the previous studies (Fig.3). The reduction of Kv4.2 and 2.1 expressions and no significant change in Kv4.3 levels in LV may suggest a severe imbalance in transient outward currents, which may be a serious complication in diabetes. Recent studies by Milstein et al. [46] showed that changes in functional expression of Kir2.1 modulates expression of Scn5a (Na_V_1.5) and vice versa to alter cardiac excitability. Interestingly, our data also showed a significant decrease in transcript levels of Scn5a as well as Scn1b in epicardium of LV, but only reduction of Scn1b in RV of db/db mice (Fig.2 & 3). This suggests alteration in cardiac excitability in diabetic heart, which may contribute to DCM. Together, our studies probing the role of ion channel expression clearly indicated their roles towards electrical remodeling in DCM.

The homeobox transcriptional factor Iroquois protein 5 (Irx5) is differentially expressed in a gradient across the left ventricle of heart [47], [48], and deletion of this gene cause arrhythmias. Costantini et al. [48] showed that Irx5 inhibits the activity of Kv4.2 promoter in a dose-dependent manner with the association of a cardiac-specific co-repressor, mBop. Our mRNA data for Irx5 in ventricle of db/db mice shows increased expression in RV suggesting a reduction of Kv4.2 expressions implicating the role of Irx5 as transcriptional repressor (Fig.2b). But, the Irx5 expression levels did not change significantly at its protein levels in RV (Fig.4). Therefore, our data suggests that the ventricular electrical remodeling in db/db mice may be regulated through some other mechanism which is independent of Irx5. Transcriptional factors such as GATA4 and GATA6 are known to be important in cardiac development and function [49], and their expression patterns in the heart resembles Kv4.2 expression, we also examined the possible regulation of these two transcriptional factors. Although our mRNA data did not show any significant change in expression of GATA4 in LV, the mRNA of GATA6 significantly reduced in both ventricles of db/db treated mice (Fig.2 & 3). Recent investigation by Jia et al. [50] showed that GATA4 produce larger increase in Kv4.2 expression via its promoter than GATA6. From these reports, it may be evident that transcriptional factors GATA4 and 6 are likely to play an important role in regulating Kv4.2 expressions in diabetic hearts.

Elevated expression and activation of the ubiquitous transcriptional factor, nuclear factor kappa B (NFκB) is reported in cardiac hypertrophy and heart diseases [51], [52], [53], [54], [55]. In a recent study, activation of NFκB decreased *I*_to,f_ by decreasing KChIP2 expression and inhibition of its activity increased both I_to,f_ and KChIP2 [23]. As we noted a significant reduction in KChIP2 gene expressions in ventricles of db/db mice, we also investigated if this effect is by the activation of NFκB. Our qRT-PCR data clearly showed a significant increase in transcripts of both NFκB1 and 2 in RV (Fig.2). In contrast a reduction in NFκB2 was observed in LV of db/db mice (Fig.3) with no alterations in NFkB1. Interestingly we observed a significant reduction of p-50 isoform of NFkB, but no change in its p-105 in LV of db/db mice (Fig.5). Also the ELISA data showed that the changes in NFkB expression in ventricles of db/db are independent of TNF-α (Fig.6). Existing reports also suggests that NFkB inhibits the expression of another muscle specific transcriptional factor Mef2c [56]. We observed a significant increase in NFkB at protein and mRNA level in the RV, along with a significant reduction (−2.8 fold) of its target Mef2c (Table.2). However, in contrast to RV we noted down-regulation of NFkB and up-regulation of Mef2c (4.8-fold) in the LV (Table.3). This clearly suggests that although NFκB is not regulating Kv4.2 expression, but may play a major role in regulating other genes and transcriptional factors in both ventricles of db/db mice and studying the molecular mechanisms would offer insights into disease pathogenicity.

To date two possible mechanisms of Kv4.2 regulation are reported: 1) Irx5 mediated repression of Kv4.2 [35], [36], [37] and 2) NFkB mediated regulation of Kv4.2 via its associated protein KChIP2 [23]. However, except Kv4.2 and KChIP2, neither of its regulators Irx5 or NFkB is commonly regulated in RV and LV of db/db hearts in our study. This further suggested the possibility that the Kv4.2 regulation in db/db may be independent of Irx5 and NFkB.

As microRNAs (miRNAs) are known to play a major role in regulating many important cellular mechanisms, we investigated the possibility of miRNA mediated regulation of Kv4.2 in diabetic condition in this study. As a first step, we utilized *in silico* tools to identify the miRNA that can potentially regulate Kv4.2 gene directly and identified miR-301a as a potential regulator of Kv4.2 (Fig.8a), which was also shown to regulate NFkB [22]. The expression of this miRNA was also investigated in RV and LV of db/db hearts by TaqMan assays and our data clearly showed a significant elevation of miR-301a (Fig.7a). We also investigated if the change in expression of this miR in diabetic condition is due to dysregulation of miRNA processing enzymes. Our qRT-PCR data in RV showed no significant change in any of the miRNA processing enzymes such as Dicer, Drosha and Exportin5 (Table 2), indicating that the diabetic condition effects the expression of miRs but not their processing from pre-miRs to mature miRNAs.

Loss of function approach in *in vitro* model (H9C2 cells) using miR-301a inhibitor was used to test if the increase in miR-301 has a role in regulation of Kv4.2 gene expression. Optimization experiments showed that inhibition of miR-301a with its inhibitor (50 nM) for 48 h is optimum, but maximum inhibition was achieved at 72 h with the additional supply of miR-301a inhibitor after 48 h (72 h‡ in Fig.7b). We also investigated the expression of Kv4.2 gene along with other genes in the cells inhibited with miR-301a. Our data showed that Kv4.2 expression was elevated significantly at 48 h by inhibition of miR-301a, without changing the expression of NFkB and Irx5 (Fig.7d). This further suggests that the miR-301a regulation of Kv4.2 may be independent of Irx5 and NFkB. Although we did not find any significant change in Kv4.2 expression both at mRNA and protein levels after 24 h of inhibitor treatment, we found significant elevation at 48 h compared to the scrambled inhibitor group (control) (Fig.8c). To identify specificity as to how miR-301a regulates Kv4.2 expressions, we utilized miRNA targeting luciferase vector system in which the miRNA binding ‘seed’ sequence was placed after the luciferase gene and luciferase activity was measured *in vitro* after the addition of miR-inhibitor or mimic. Our data showed that addition of miR-inhibitor to the cells transfected with miR-301a ‘seed’ sequence significantly increased luciferase activity, whereas its activity was significantly reduced in the cells added with miR-mimic compared with the scrambled sequence (Fig.8b). From this data it is evident that the regulation of Kv4.2 expression by miR-301a is by direct binding and is independent of Irx5 and NFkB.

To evaluate the overall changes in the potassium channel expression and activity due to miR-301a inhibitor, we treated the H9C2 cells with miR-301a inhibitor and patched the cells at 72 h time point for recording the outward currents. This assay allowed us to identify changes caused by attenuating miR-301a and affect functional activity of the potassium channels. Potassium currents in the H9C2 cells were measured from scrambled or miR-301a inhibitor treated cells. We demonstrate that treatment with miR-301a inhibitor significantly accelerated the inactivation τ (tau) suggesting that miR-301a inhibitor caused an overall change in the ion channel expression in the *in vitro* cell culture model. These experiments attest to the possibility that miR-301a inhibitor treatment causes improved activity of potassium currents at functional level and validated the expression studies (Fig 9).

**Figure 10:**
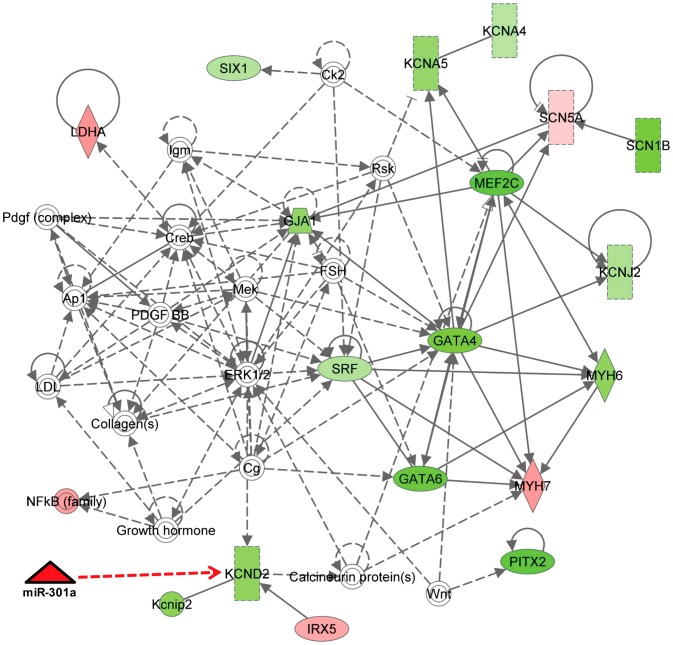
Cellular pathway affecting the cardiac arrhythmia, cardiovascular disease and tachycardia in RV of diabetic hearts. The differentially regulated genes in qRT-PCR analysis in right ventricle of db/db hearts compared to their wild type controls were taken as input data and Ingenuity Pathway Analysis (IPA) software was used to build interactive network(s) based on the relations available from its library. Genes shown in green color were down-regulated and up-regulated genes were shown in red color, where intensity of color is proportional to the fold values. Dotted arrows represent indirect relations and solid lines represent direct relations between the genes. Genes that are present in the network but not present in the input data are shown without any color.

To develop an overall understanding of our findings at the molecular level in diabetic heart, we utilized Ingenuity pathway analysis (IPA) and provided our experimental data as input for predicting possible pathways that are involved in the remodeling. Based on this analysis the pathways regulated in the diabetic right ventricle suggest that Kv4.2 (kcnd2) along with GATA4, MHCα, MHCβ and Scn5a are majorly altered (Fig 10). Analysis network eligible molecules (selected genes within the desired cut-off limits) serve as “seeds” for generating networks by IPA and Ingenuity knowledge base maximize their connectivity based on existing literature. IPA also generates canonical pathways based on available literature from its library [57]. As expected, IPA also suggests the direct relation between Kv4.2 expression with its chaperon KChIP2 and its repressor Irx5 (Fig.10). The IPA also suggests that Kv1.4 and Kv1.5 can form stable heterodimers in physiological conditions [58]. The network also suggests that heart specific transcriptional factor; serum response factor (Srf) affects the expression of GATA4 and MHCα & β significantly, without affecting Mef2c and GATA6 [59]. Although with low significance, our data also showed down-regulation of Srf in RV, along with significantly down-regulated GATA4 and MHC α & β expression. Based on our *in vitro* and *in vivo* studies on Kv4.2 regulation by miR-301a, we have added novel information to the IPA network and added a new relation to the existing network (Fig. 10). In addition, IPA also suggested cardiac arrhythmia, cardiovascular disease, and tachycardia network pathway is the top most networks associated with the genes that are differentially regulated in RV of db/db mice (data not shown).

## Conclusions

Overall, the study finds the role of key transcriptional mediators and the direct involvement of miR-301a in Kv4.2 channel modulation in diabetes. The central role of miR-301a in Kv4.2 regulation brings new understanding that is previously not reported in diabetes. The ion channel based remodeling with decreased repolarization reserve and ion channel disruption in diabetes occurs in a regional specific manner in which the right and the left ventricles remodel using distinct molecular pathways. The role of KChIP2 and NFkB was studied with a focus to elucidate the molecular detail in which we illustrate that the NFkB is significantly up regulated in the RV whereas in the left ventricle it is unchanged. The data presented here clearly shows that Kv4.2 modulation is independent of Irx5 and therefore microRNA mediated regulation may be important for better understanding of diabetic cardiomyopathy. We also report the differential regulation of KChIP2 in diabetic heart suggesting the role of this chaperon in Kv4.2 regulation.

## References

[1] Haffner SM, Lehto S, Ronnemaa T, Pyorala K, Laakso M (1998) Mortality from coronary heart disease in subjects with type 2 diabetes and in nondiabetic subjects with and without prior myocardial infarction. N Engl J Med 339: 229–234. 9673301 10.1056/NEJM199807233390404

[2] Yeung CY, Lam KS, Li SW, Lam KF, Tse HF, et al.. (2012) Sudden Cardiac Death After Myocardial Infarction in Type 2 Diabetic Patients With No Residual Myocardial Ischemia. Diabetes Care. 10.2337/dc12-0118 PMC3507604 22875229

[3] Casis O, Gallego M, Iriarte M, Sanchez-Chapula JA (2000) Effects of diabetic cardiomyopathy on regional electrophysiologic characteristics of rat ventricle. Diabetologia 43: 101–109. 10672450 10.1007/s001250050013

[4] Li X, Xu Z, Li S, Rozanski GJ (2005) Redox regulation of Ito remodeling in diabetic rat heart. Am J Physiol Heart Circ Physiol 288: H1417–1424. 15539426 10.1152/ajpheart.00559.2004

[5] Nishiyama A, Ishii DN, Backx PH, Pulford BE, Birks BR, et al (2001) Altered K(+) channel gene expression in diabetic rat ventricle: isoform switching between Kv4.2 and Kv1.4. American journal of physiology Heart and circulatory physiology 281: H1800–1807. 11557574 10.1152/ajpheart.2001.281.4.H1800

[6] Heather LC, Clarke K (2011) Metabolism, hypoxia and the diabetic heart. Journal of molecular and cellular cardiology 50: 598–605. 21262230 10.1016/j.yjmcc.2011.01.007

[7] Fein FS (1990) Diabetic cardiomyopathy. Diabetes care 13: 1169–1179. 2261838 10.2337/diacare.13.11.1169

[8] Fein FS, Aronson RS, Nordin C, Miller-Green B, Sonnenblick EH (1983) Altered myocardial response to ouabain in diabetic rats: mechanics and electrophysiology. Journal of molecular and cellular cardiology 15: 769–784. 6361268 10.1016/0022-2828(83)90336-x

[9] Malhotra A, Penpargkul S, Fein FS, Sonnenblick EH, Scheuer J (1981) The effect of streptozotocin-induced diabetes in rats on cardiac contractile proteins. Circulation research 49: 1243–1250. 6458419 10.1161/01.res.49.6.1243

[10] Khavandi K, Khavandi A, Asghar O, Greenstein A, Withers S, et al (2009) Diabetic cardiomyopathy--a distinct disease? Best practice & research Clinical endocrinology & metabolism 23: 347–360. 19520308 10.1016/j.beem.2008.10.016

[11] Hu S, Huang M, Li Z, Jia F, Ghosh Z, et al (2010) MicroRNA-210 as a novel therapy for treatment of ischemic heart disease. Circulation 122: S124–131. 20837903 10.1161/CIRCULATIONAHA.109.928424 PMC2952325

[12] van Rooij E, Sutherland LB, Thatcher JE, DiMaio JM, Naseem RH, et al (2008) Dysregulation of microRNAs after myocardial infarction reveals a role of miR-29 in cardiac fibrosis. Proceedings of the National Academy of Sciences of the United States of America 105: 13027–13032. 18723672 10.1073/pnas.0805038105 PMC2529064

[13] Yang B, Lin H, Xiao J, Lu Y, Luo X, et al (2007) The muscle-specific microRNA miR-1 regulates cardiac arrhythmogenic potential by targeting GJA1 and KCNJ2. Nature medicine 13: 486–491. 10.1038/nm1569 17401374

[14] Veglio M, Chinaglia A, Cavallo-Perin P (2004) QT interval, cardiovascular risk factors and risk of death in diabetes. Journal of endocrinological investigation 27: 175–181. 15129815 10.1007/BF03346265

[15] Qin D, Huang B, Deng L, El-Adawi H, Ganguly K, et al (2001) Downregulation of K(+) channel genes expression in type I diabetic cardiomyopathy. Biochemical and biophysical research communications 283: 549–553. 11341759 10.1006/bbrc.2001.4825

[16] Guo W, Jung WE, Marionneau C, Aimond F, Xu H, et al (2005) Targeted deletion of Kv4.2 eliminates I(to,f) and results in electrical and molecular remodeling, with no evidence of ventricular hypertrophy or myocardial dysfunction. Circulation research 97: 1342–1350. 16293790 10.1161/01.RES.0000196559.63223.aa

[17] Barry DM, Xu H, Schuessler RB, Nerbonne JM (1998) Functional knockout of the transient outward current, long-QT syndrome, and cardiac remodeling in mice expressing a dominant-negative Kv4 alpha subunit. Circulation research 83: 560–567. 9734479 10.1161/01.res.83.5.560

[18] Lugo JN, Brewster AL, Spencer CM, Anderson AE (2012) Kv4.2 knockout mice have hippocampal-dependent learning and memory deficits. Learning & memory 19: 182–189. 22505720 10.1101/lm.023614.111 PMC3348517

[19] Yuan W, Burkhalter A, Nerbonne JM (2005) Functional role of the fast transient outward K+ current IA in pyramidal neurons in (rat) primary visual cortex. The Journal of neuroscience: the official journal of the Society for Neuroscience 25: 9185–9194. 16207878 10.1523/JNEUROSCI.2858-05.2005 PMC6725755

[20] van Rooij E, Marshall WS, Olson EN (2008) Toward microRNA-based therapeutics for heart disease: the sense in antisense. Circulation research 103: 919–928. 18948630 10.1161/CIRCRESAHA.108.183426 PMC2725407

[21] Zhao Y, Ransom JF, Li A, Vedantham V, von Drehle M, et al (2007) Dysregulation of cardiogenesis, cardiac conduction, and cell cycle in mice lacking miRNA-1-2. Cell 129: 303–317. 17397913 10.1016/j.cell.2007.03.030

[22] Lu Z, Li Y, Takwi A, Li B, Zhang J, et al (2011) miR-301a as an NF-kappaB activator in pancreatic cancer cells. The EMBO journal 30: 57–67. 21113131 10.1038/emboj.2010.296 PMC3020116

[23] Panama BK, Latour-Villamil D, Farman GP, Zhao D, Bolz SS, et al (2011) Nuclear factor kappaB downregulates the transient outward potassium current I(to,f) through control of KChIP2 expression. Circulation research 108: 537–543. 21252158 10.1161/CIRCRESAHA.110.229112

[24] Feng B, Chen S, George B, Feng Q, Chakrabarti S (2010) miR133a regulates cardiomyocyte hypertrophy in diabetes. Diabetes/metabolism research and reviews 26: 40–49. 20013939 10.1002/dmrr.1054

[25] Panguluri SK, Kakar SS (2009) Effect of PTTG on endogenous gene expression in HEK 293 cells. BMC genomics 10: 577. 19958546 10.1186/1471-2164-10-577 PMC2793268

[26] Tipparaju SM, Li XP, Kilfoil PJ, Xue B, Uversky VN, et al (2012) Interactions between the C-terminus of Kv1.5 and Kvbeta regulate pyridine nucleotide-dependent changes in channel gating. Pflugers Archiv: European journal of physiology 463: 799–818. 22426702 10.1007/s00424-012-1093-z PMC3367765

[27] Panguluri SK, Bhatnagar S, Kumar A, McCarthy JJ, Srivastava AK, et al (2010) Genomic profiling of messenger RNAs and microRNAs reveals potential mechanisms of TWEAK-induced skeletal muscle wasting in mice. PloS one 5: e8760. 20098732 10.1371/journal.pone.0008760 PMC2808241

[28] Zhang H, Cuevas J (2005) sigma Receptor activation blocks potassium channels and depresses neuroexcitability in rat intracardiac neurons. The Journal of pharmacology and experimental therapeutics 313: 1387–1396. 15764734 10.1124/jpet.105.084152

[29] Semeniuk LM, Kryski AJ, Severson DL (2002) Echocardiographic assessment of cardiac function in diabetic db/db and transgenic db/db-hGLUT4 mice. American journal of physiology Heart and circulatory physiology 283: H976–982. 12181126 10.1152/ajpheart.00088.2002

[30] Bhatnagar S, Panguluri SK, Gupta SK, Dahiya S, Lundy RF, et al (2010) Tumor necrosis factor-alpha regulates distinct molecular pathways and gene networks in cultured skeletal muscle cells. PloS one 5: e13262. 20967264 10.1371/journal.pone.0013262 PMC2953497

[31] Fernandez-Velasco M, Ruiz-Hurtado G, Hurtado O, Moro MA, Delgado C (2007) TNF-alpha downregulates transient outward potassium current in rat ventricular myocytes through iNOS overexpression and oxidant species generation. American journal of physiology Heart and circulatory physiology 293: H238–245. 17337591 10.1152/ajpheart.01122.2006

[32] Petkova-Kirova PS, Gursoy E, Mehdi H, McTiernan CF, London B, et al (2006) Electrical remodeling of cardiac myocytes from mice with heart failure due to the overexpression of tumor necrosis factor-alpha. American journal of physiology Heart and circulatory physiology 290: H2098–2107. 16339842 10.1152/ajpheart.00097.2005

[33] Kawada H, Niwano S, Niwano H, Yumoto Y, Wakisaka Y, et al (2006) Tumor necrosis factor-alpha downregulates the voltage gated outward K+ current in cultured neonatal rat cardiomyocytes: a possible cause of electrical remodeling in diseased hearts. Circulation journal: official journal of the Japanese Circulation Society 70: 605–609. 16636498 10.1253/circj.70.605

[34] Shimoni Y, Liu XF (2003) Role of PKC in autocrine regulation of rat ventricular K+ currents by angiotensin and endothelin. American journal of physiology Heart and circulatory physiology 284: H1168–1181. 12626328 10.1152/ajpheart.00748.2002

[35] Kuo HC, Cheng CF, Clark RB, Lin JJ, Lin JL, et al (2001) A defect in the Kv channel-interacting protein 2 (KChIP2) gene leads to a complete loss of I(to) and confers susceptibility to ventricular tachycardia. Cell 107: 801–813. 11747815 10.1016/s0092-8674(01)00588-8

[36] Costantini DL, Arruda EP, Agarwal P, Kim KH, Zhu Y, et al (2005) The homeodomain transcription factor Irx5 establishes the mouse cardiac ventricular repolarization gradient. Cell 123: 347–358. 16239150 10.1016/j.cell.2005.08.004 PMC1480411

[37] He W, Jia Y, Takimoto K (2009) Interaction between transcription factors Iroquois proteins 4 and 5 controls cardiac potassium channel Kv4.2 gene transcription. Cardiovascular research 81: 64–71. 18815185 10.1093/cvr/cvn259 PMC2721642

[38] Shimoni Y, Chuang M, Abel ED, Severson DL (2004) Gender-dependent attenuation of cardiac potassium currents in type 2 diabetic db/db mice. The Journal of physiology 555: 345–354. 14694146 10.1113/jphysiol.2003.055590 PMC1664833

[39] Radicke S, Cotella D, Graf EM, Banse U, Jost N, et al (2006) Functional modulation of the transient outward current Ito by KCNE beta-subunits and regional distribution in human non-failing and failing hearts. Cardiovascular research 71: 695–703. 16876774 10.1016/j.cardiores.2006.06.017

[40] Gidh-Jain M, Huang B, Jain P, el-Sherif N (1996) Differential expression of voltage-gated K+ channel genes in left ventricular remodeled myocardium after experimental myocardial infarction. Circulation research 79: 669–675. 8831490 10.1161/01.res.79.4.669

[41] Kaprielian R, Wickenden AD, Kassiri Z, Parker TG, Liu PP, et al (1999) Relationship between K+ channel down-regulation and [Ca2+]i in rat ventricular myocytes following myocardial infarction. The Journal of physiology 517 (Pt1): 229–245. 10226162 10.1111/j.1469-7793.1999.0229z.x PMC2269317

[42] An WF, Bowlby MR, Betty M, Cao J, Ling HP, et al (2000) Modulation of A-type potassium channels by a family of calcium sensors. Nature 403: 553–556. 10676964 10.1038/35000592

[43] Wang Y, Xu H, Kumar R, Tipparaju SM, Wagner MB, et al (2003) Differences in transient outward current properties between neonatal and adult human atrial myocytes. Journal of molecular and cellular cardiology 35: 1083–1092. 12967631 10.1016/s0022-2828(03)00200-1

[44] Mariappan N, Elks CM, Sriramula S, Guggilam A, Liu Z, et al (2010) NF-kappaB-induced oxidative stress contributes to mitochondrial and cardiac dysfunction in type II diabetes. Cardiovascular research 85: 473–483. 19729361 10.1093/cvr/cvp305 PMC2860708

[45] Yue P, Arai T, Terashima M, Sheikh AY, Cao F, et al (2007) Magnetic resonance imaging of progressive cardiomyopathic changes in the db/db mouse. American Journal of Physiology - Heart and Circulatory Physiology 292: H2106–H2118. 17122193 10.1152/ajpheart.00856.2006

[46] Milstein ML, Musa H, Balbuena DP, Anumonwo JMB, Auerbach DS, et al.. (2012) Dynamic reciprocity of sodium and potassium channel expression in a macromolecular complex controls cardiac excitability and arrhythmia. Proceedings of the National Academy of Sciences. 10.1073/pnas.1109370109 PMC3412015 22509027

[47] Rosati B, Grau F, McKinnon D (2006) Regional variation in rnRNA transcript abundance within the ventricular wall. Journal of molecular and cellular cardiology 40: 295–302. 16412459 10.1016/j.yjmcc.2005.11.002

[48] Costantini DL, Arruda EP, Agarwal P, Kim KH, Zhu YH, et al (2005) The homeodomain transcription factor lrx5 establishes the mouse cardiac ventricular repolarization gradient. Cell 123: 347–358. 16239150 10.1016/j.cell.2005.08.004 PMC1480411

[49] Laverriere AC, MacNeill C, Mueller C, Poelmann RE, Burch JB, et al (1994) GATA-4/5/6, a subfamily of three transcription factors transcribed in developing heart and gut. The Journal of biological chemistry 269: 23177–23184. 8083222

[50] Jia Y, Takimoto K (2003) GATA and FOG2 transcription factors differentially regulate the promoter for Kv4.2 K+ channel gene in cardiac myocytes and PC12 cells. Cardiovascular research 60: 278–287. 14613857 10.1016/s0008-6363(03)00528-5

[51] Wong SC, Fukuchi M, Melnyk P, Rodger I, Giaid A (1998) Induction of cyclooxygenase-2 and activation of nuclear factor-kappaB in myocardium of patients with congestive heart failure. Circulation 98: 100–103. 9679714 10.1161/01.cir.98.2.100

[52] Purcell NH, Tang G, Yu C, Mercurio F, DiDonato JA, et al (2001) Activation of NF-kappa B is required for hypertrophic growth of primary rat neonatal ventricular cardiomyocytes. Proceedings of the National Academy of Sciences of the United States of America 98: 6668–6673. 11381115 10.1073/pnas.111155798 PMC34410

[53] Higuchi Y, Otsu K, Nishida K, Hirotani S, Nakayama H, et al (2002) Involvement of reactive oxygen species-mediated NF-kappa B activation in TNF-alpha-induced cardiomyocyte hypertrophy. Journal of molecular and cellular cardiology 34: 233–240. 11851362 10.1006/jmcc.2001.1505

[54] Gupta S, Young D, Maitra RK, Gupta A, Popovic ZB, et al (2008) Prevention of cardiac hypertrophy and heart failure by silencing of NF-kappaB. Journal of molecular biology 375: 637–649. 18037434 10.1016/j.jmb.2007.10.006 PMC2277468

[55] Gupta S, Young D, Sen S (2005) Inhibition of NF-kappaB induces regression of cardiac hypertrophy, independent of blood pressure control, in spontaneously hypertensive rats. American journal of physiology Heart and circulatory physiology 289: H20–29. 15749748 10.1152/ajpheart.00082.2005

[56] Kumar A, Lin Z, SenBanerjee S, Jain MK (2005) Tumor necrosis factor alpha-mediated reduction of KLF2 is due to inhibition of MEF2 by NF-kappaB and histone deacetylases. Molecular and cellular biology 25: 5893–5899. 15988006 10.1128/MCB.25.14.5893-5903.2005 PMC1168833

[57] Naga Prasad SV, Duan ZH, Gupta MK, Surampudi VS, Volinia S, et al (2009) Unique microRNA profile in end-stage heart failure indicates alterations in specific cardiovascular signaling networks. The Journal of biological chemistry 284: 27487–27499. 19641226 10.1074/jbc.M109.036541 PMC2785678

[58] Nitabach MN, Llamas DA, Araneda RC, Intile JL, Thompson IJ, et al (2001) A mechanism for combinatorial regulation of electrical activity: Potassium channel subunits capable of functioning as Src homology 3-dependent adaptors. Proceedings of the National Academy of Sciences of the United States of America 98: 705–710. 11149959 10.1073/pnas.031446198 PMC14652

[59] Balza RO Jr, Misra RP (2006) Role of the serum response factor in regulating contractile apparatus gene expression and sarcomeric integrity in cardiomyocytes. The Journal of biological chemistry 281: 6498–6510. 16368687 10.1074/jbc.M509487200

